# New mechanism for mesenchymal stem cell microvesicle to restore lung permeability: intracellular S1P signaling pathway independent of S1P receptor-1

**DOI:** 10.1186/s13287-022-03177-4

**Published:** 2022-10-08

**Authors:** Lifang Ye, Jieqiong Song, Yijun Zheng, Ming Zhong, Jun Liu, Duming Zhu, Shuling Hu

**Affiliations:** 1 Department of Intensive Care Medicine, Medical School, Zhongshan Hospital, Fudan University, 180 Fenglin Road, Xuhui District, Shanghai, 200032 China; 2 Department of Intensive Care Medicine, Gusu School, Suzhou Hospital, Nanjing Medical University, No 16 West Baita Road, Suzhou, 215001 China; 3 Department of Critical Care Medicine, Zhongshan Hospital, Fudan University, Shanghai, China

**Keywords:** Mesenchymal stem cell, Microvesicles, Human lung microvascular endothelial cell, Pulmonary endothelial permeability, Sphingosine-1-phosphate

## Abstract

**Background:**

Microvesicles (MVs) derived from human bone marrow mesenchymal stem cell (MSC) were demonstrated to restore lung protein permeability and attenuate acute lung injury. In our previous study, we found that MSC MV increased sphingosine-1-phosphate (S1P) kinase1 mRNA levels in injured human lung microvascular endothelial cells (HLMVEC) significantly. However, the role of S1P signaling in MSC MV to restore lung protein permeability is unknown.

**Methods:**

In this study, we hypothesized that MSC MV might restore lung permeability in part through increasing intracellular S1P signaling pathway in injured HLMVEC independent of S1P receptors. We used the transwell co-culture system to study the effect of MSC MV on protein permeability of Lipopolysaccharide (LPS) damaged HLMVEC.

**Results:**

Our results showed that LPS significantly increased the permeability of HLMVEC to FITC-dextran (70 kDa) within 24 h. MSC MV restores this permeability and, to a large extent, prevents the cytoskeleton protein F-actin from recombining into “actin stress fibers,” and restores the positions of tight junctions and adhesion junctions in the damaged HLMVEC. This therapeutic effect of MSC MV was related to the increase in the S1P level in injured HLMVEC and was not eliminated when adding the antagonist of S1P receptor, suggesting that MSC MV to restore lung permeability was independent of S1P receptors on HLMVEC. Laser confocal further observed that Ca^2+^ mobilization and Rac1 activation in LPS injured HLMVEC were increased in parallel with the increase in intracellular S1P level after MSC MV treatment.

**Conclusions:**

In short, MSC MV partially restored protein permeability across HLMVEC through the intracellular S1P signaling pathway independent of S1P receptor-1.

## Introduction

Acute respiratory distress syndrome (ARDS) is characterized by diffuse injury of pulmonary capillary endothelium and alveolar epithelium caused by a variety of causes including severe infection, trauma, shock and blood transfusion [1, 2]. The clinical manifestations include progressive dyspnea and refractory hypoxemia [3]. ARDS is one of the most important causes of death of critically ill patients in the intensive care unit (ICU) and a major cause of poor prognosis in critically ill patients. The researches on ARDS have been deepened and great progresses have been made in clinical treatment in recent years. In particular, treatment strategies such as low tidal volume mechanical ventilation [4], fluid restriction, prone position ventilation [5] and muscle relaxants [6] have been proved to reduce the mortality of such patients to a certain extent. However, ARDS mortality is still as high as 40% [1, 7]. Therefore, to explore innovative and effective treatments of ARDS is imminent.

Mesenchymal stem cell (MSC)-based therapy for the treatment of acute lung injury is very attractive. Evidence has shown that the administration of MSC improved ARDS, whether from endotoxin, or live* E. coli* bacteria. Recently, MSC has been found to release microvesicles (MSC MV), which are anuclear particles, 50 nm to 1 µm in size, and contain numerous bioactive materials, such as proteins and lipids, mitochondria, as well as nucleic acid material in the form of DNA, mRNA, microRNA (miRNA) and noncoding RNA [8–10]. Microvesicles were originally described as the elimination of unwanted compounds from cells and considered as a means of releasing debris from cells [11]. Now, microvesicles have garnered increasing interest from basic and clinical. They are considered as important intercellular communication media that can participate in cell-to-cell communication [8, 12–16] and transfer of cellular material [13, 14, 16, 17].

Studies have demonstrated that MSC MV can home to the inflammatory site as its parent cells and transfer the bioactive molecular to promote growth, angiogenesis, anti-apoptosis, anti-oxidation, metabolism and immunoregulatory properties in injured tissues [18–20]. Our previous and foreign studies have found that MSC MV can significantly reduce pulmonary edema in ARDS mice, control lung inflammation and reduce mortality in mice [17, 21]. In the vitro study, we demonstrated that MSC MV restored protein permeability across injured HLMVEC partially through increasing Ang1 secretion in injured HLMVEC. Moreover, we found that MSC MV increased sphingosine-1-phosphate (S1P) kinase1 mRNA levels significantly, which indicates that S1P signaling may also play a role in the restoration of endothelial permeability by MSC MV [22]. In this current study, we aimed to explore the role of S1P signaling way in restoration the protein permeability across the injured HLMVEC monolayer and its underlying mechanism.

## Materials and methods

### Mesenchymal stem cells

Human BMSCs were purchased from Promocell (Promocell, C-12974, Germany, www.promocell.com). The MSCs were isolated from bone marrow of healthy donors. Cells are routinely identified and meet the standard of the International Society of Cellular Therapy. MSCs were cultured in Mesenchymal Stem Cell Growth Medium 2 (Promocell, C-28009, Germany, www.promocell.com) supplemented with human epidermal growth factor, vascular endothelial growth factor, R3-insulin-like growth factor-1, ascorbic acid, hydrocortisone, human fibroblast growth factor-beta, 5% fetal bovine serum, 1% penicillin/streptomycin and maintained in a humidified incubator with 5% CO_2_ at 37 °C. Change the medium every 2–3 days. Passage the cells when they reach 90% confluence. In the experiment, MSCs with a total passage number of < 10 were used.

### Isolation of MV

As mentioned earlier, we used ultracentrifugation to isolate MV from the conditioned medium of human bone marrow-derived MSCs [21]. In short, MSCs were serum starved in a conditioned medium (a-MEM supplemented with 0.5% bovine albumin fraction [www.sigmaaldrich.com]). After 48 h, collect the conditioned medium, centrifuge at 3000 rpm for 20 min to remove cell debris, then centrifuge at 100,000 g (Beckman Coulter Optima L-100XP ultracentrifuge) at 4 °C for 1 h, aspirate the supernatant and wash it with phosphate-buffered saline (PBS) and centrifuge again at 4 °C at 100,000 g for 1 h. The pellet containing MV was resuspended in PBS and stored at − 80 °C. Ten microliters of MV is equivalent to the MV released by 1 million MSCs.

### MSC MV characterization

Human MSC monolayers grown on glass coverslips or isolated MSC MV were fixed with 3% (wt/vol) karnovsky fixative for 2 h at 4 °C. The monolayers were post fixed for 2 h in 1% veronal-buffered osmic acid and then dehydrated in graded ethanol and/or propylene oxide. The cell preparations were then embedded in epon or araldite resins cured at 60 °C. Thin sections were contrasted with saturated aqueous uranyl acetate and Reynolds lead citrate. The sections were then imaged with a JEOL 1200 EX transmission electron microscope operating at 80 kV. The phenotypic profile of MSC MV obtained was also determined by western blot. The particles obtained after ultracentrifugation are also tracked and analyzed by Nanoparticle Tracking Analysis (NTA) to analyze the diameter distribution and concentration of the microvesicles.

### Primary cultures of HLMVEC

HLMVEC were obtained from small vessels within normal lung tissue (Promocell,C-12281 Germany, www.promocell.com). HLMVEC were cultured in endothelial cell growth medium (EBM2 basal medium supplemented with human epidermal growth factor, vascular endothelial growth factor, R3-insulin-like growth factor-1, ascorbic acid, hydrocortisone, human fibroblast growth factor-beta, 5% fetal bovine serum, gentamicin/amphotericin-B and 1% penicillin/streptomycin [Promocell, C-22022,Germany, www.promocell.com]) and incubated in a humidified incubator with 5% CO_2_ at 37 °C. Change the growth medium 24 h after inoculation, and every other day thereafter. When the cells reach 70–85% confluence, they are subcultured. HLMVEC with a total passage number of < 9 were used in all experiments.

### Coculture of LPS-stimulated HLMVEC and MSC MV

We used a transwell coculture system (0.4-mm pore size and collagen I-coated, Costar, Corning, Tewksbury, MA, www.corning.com) to study the effects of MSC MV on the protein permeability across HLMVEC monolayer injured by LPS (500 ng/ml) as a substitute for ALI pulmonary edema fluid. The transwell inserts were placed in a 24-well plates. HLMVEC were seeded in the inserts at a density of 2 × 10^5^ cells/insert and maintained in a humidified incubator with 5% CO_2_ at 37 °C to form a monolayer. After 24 h, the HLMVEC monolayer was exposed to LPS or LPS with MSC MV at 60 ul with or without W123 which was the sphingosine-1-phosphate receptor-1 (S1PR1) antagonist. After 24 h, the culture medium in the inserts was aspirated and replaced with 100 ml fresh culture medium contained FITC-dextran (100 ug/ml, 70 kDa, www. sigmaaldrich. com) into upper compartment. Cells were maintained in incubator for 1 h. Then, 100 ul culture mediums were obtained from the upper compartment and the lower compartment, respectively. We used a plate reader (FlexStation 3: FV05706) to measure the fluorescence in the medium and calculate the one-way flux of FITC-dextran from the upper chamber to the lower chamber as the protein permeability.

### Immunofluorescence microscopy

HLMVEC was inoculated in cell slices, maintained in a humidified incubator with 5% CO_2_ at 37 ℃ to form a monolayer. Then, the cell was exposed to LPS or LPS with MSC MV or LPS with MSC MV and W123 for 24 h. The cells were washed 3 times with PBS and fixed in 4% paraformaldehyde for 15 min. Then, the cells were washed 3 times with PBS for 3 min and permeabilized with 0.1% Triton X-100 for 15 min. Wash with PBS three times for 3 min, then block with 1% BSA at room temperature for 30 min, and then incubate with primary antibody. Phalloidin (1: 200) diluted in PBS to stain the cytoskeleton for 30 min at room temperature. VE-cadherin (1: 400) diluted in PBS to stain adherens junction protein at 37 °C for 2 h. ZO-1 (1:50) diluted in PBS to stain tight junction protein at 37 °C for 2 h. Then, the cell slides were washed 3 times with PBS followed by incubation with secondary antibodies-Goat anti-rabbit IgG(H + L) conjugated with Alexa fluor®488(1:200 diluted in PBS) and Goat anti-mouse IgG(H + L) conjugated with Alexa fluor-488 (1:200 diluted in PBS) for 1 h at 37 ℃. Then, the cell slides were wash 3 times with PBS again. Finally, mount with DAPI-containing mounts. Images were obtained by confocal microscopy (FV3000, Japan).

### S1P levels detection by ELISA

HLMVEC was seeded in a 12-well plate at a density of 1 million cells per well and kept in an incubator overnight. The cells were then exposed to 500 ng/ml LPS with or without MSC MV or MSC MV plus W123. After 24 h, the supernatant was collected and stored at − 80 °C. Lyse the cells, collect the cell lysate and store at − 80 °C. We used the ELISA kit (JL14111-96 T,http://m.jonln.com) to detect the S1P levels in both the supernatants and cell lysates according to the manufacturer’s instruction.

### Measurement of intracellular Ca^2+^ concentration by confocal microscope

HLMVEC are grown in a confocal special culture dish, grown to 80–90% confluent. Then, the cells were exposed to 500 ng/ml LPS with or without MSC MV or MSC MV plus W123. After 24 h, the medium was removed and fresh endothelial medium was added containing 1umol/l Fluo-3AM (Beyotime biotechnology) to incubate at 37 °C for 15 min and then washed twice with D-PBS, and fresh medium was added and incubated at 37 °C for 20 min. Images were acquired through a confocal microscope and analyzed with ImageJ software (FV3000, Japan).

### Western blot analyses

After treatment, HLMVECs were collected and lysed with RIPA (P0013B, Beyotime biotechnology) containing 1 mmol/l PMSF (Beyotime biotechnology)) to obtain total cell protein, and then, the protein concentration of the samples was measured by BCA method according the manufacturer’s instructions (P0010, Beyotime biotechnology). The proteins were separated by 8–10% SDS-PDGE and transferred to PVDF membranes. Then, PVDF membranes were blocked in TBST containing 5% skim milk for 1 h at room temperature followed by primary antibodies for VE-cadherin (1:1000 dilution, Cell Signaling), ZO-1 (1:1000, Proteintech), Rac1 (1:1000, Proteintech), β-catenin (1:1000, Arizo), SPHK (1:500, HuaAn Biotechnology, Hangzhou, China), CD63 (1:1000, Abcam), TSG101 (1:1000, Abcam) or β-actin (1:1000, Santa Cruz, USA) as loading control at 4 °C overnight. Membranes were washed three times in TBST and then incubated in peroxidase-conjugated secondary antibody (Beijing Biodragon Immunotechnologies Co., Ltd).

### Statistical analysis

All experimental groups were repeated at least three times, each in triplicate. The data are shown as mean ± SD. For comparisons between two groups, an unpaired t test was used. For comparisons between multiple groups, analysis of variance (ANOVA) with post hoc Tukey HSD test was used. A value of *p* < *0.05* was considered statistically significant. Use GraphPad prism 6 for analysis.

## Results

### The characterization of MSC derived microvesicles

**Fig. 1:**
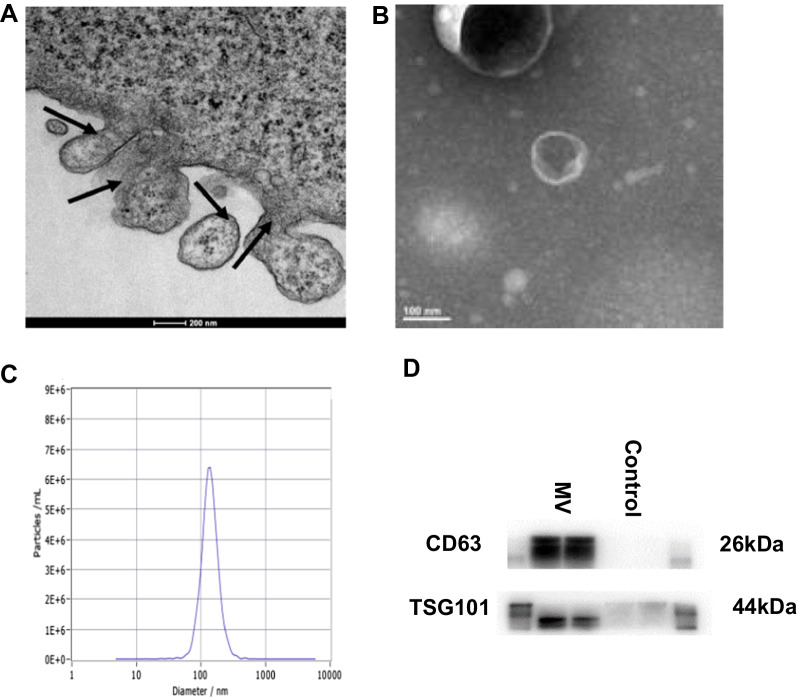
The characterization of MSC MV. MSCs were treated by serum starvation for 48 h. **A** Scanning electron microscopy showed that there were microvesicles sprouting and falling off, and the shape was like a round, vesicle-like around the surface of the starved MSCs. **B** Microvesicles were collected by ultracentrifugation. Scanning electron microscope showed that microvesicles were in the form of vesicles and had a double-layer membrane structure. **C** NTA analysis of the obtained microvesicles found that 99.2% of the microvesicles were about 136.9 nm in size, and the concentration was about 1.9 × 10^11^ per ml. **D** Western blot analysis on the expression of the MV markers CD63 and TSG101. All experiments were repeated three times. MSC, mesenchymal stem cell; MV, microvesicle; NTA, nanoparticles tracking technology

MSC MV was characterized by scanning electron microscopy, nanoparticles tracking technology analysis and western blotting. Scanning electron microscopy showed that there were microvesicles sprouting and falling off, and the shape was like a round, vesicle-like around the surface of the starved MSC (Fig. 1A). After ultracentrifugation, scanning electron microscopy showed that the extracted microvesicles were spherical particles and had a double-layer membrane structure (Fig. 1B). Nanoparticles tracking technology analysis of the obtained microvesicles found that 99.2% of the particles was around 136.9 nm in size, and the concentration was 1.9 × 10^11^ per ml (Fig. 1C). Western blotting results showed that the microvesicles expressed the MSC MV surface protein marker CD63 and TSG101 (Fig. 1D). The results implied that the collected particles were MSC MV.

### LPS increased protein permeability across HLMVEC

**Fig. 2:**
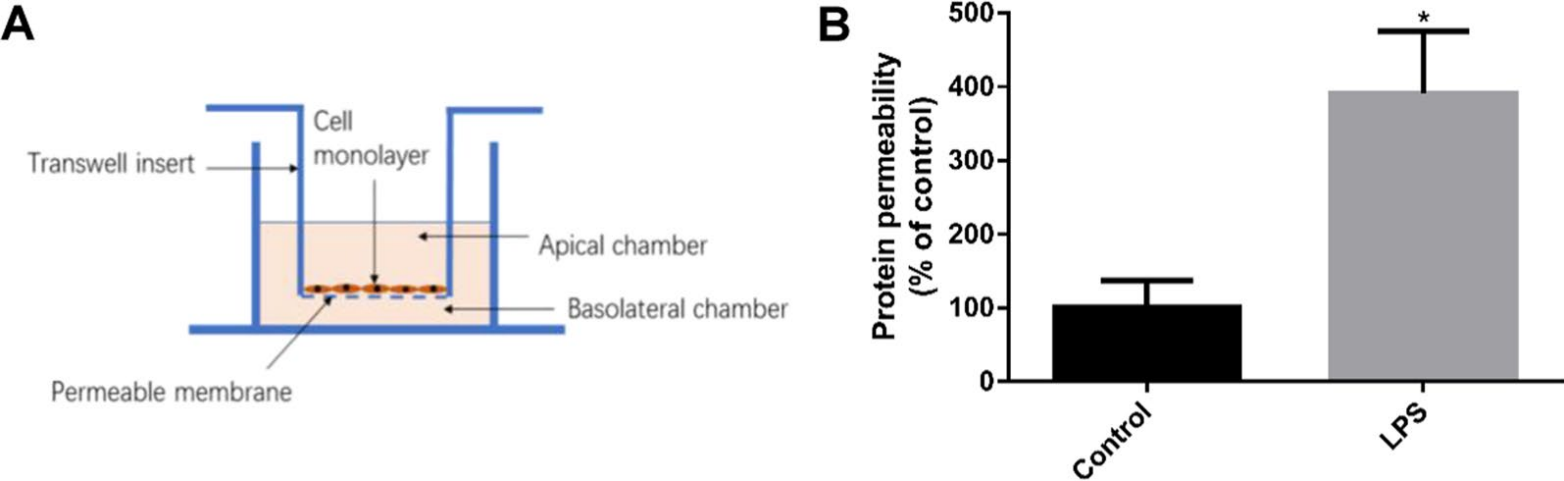
LPS increased protein permeability across the monolayer of human lung microvascular endothelial cells. **A** Transwell co-cultivation system structure diagram: HLMVEC were cultured in the apical chamber. Medium containing 500 ng/ml LPS, usually used as a substitute for acute lung injury pulmonary edema fluid, is added to the upper and lower chambers. The liquid level between the upper and lower compartments is kept equal to prevent pressure differences between the compartments. **B** LPS increased the permeability of HLMVEC monolayer by more than 3 times over 24 h. Data are presented as mean ± SD, *N* = 9; *p < 0.05 versus control using unpaired two-tailed * t* test. HLMVEC, human lung microvascular endothelial cell; LPS, lipopolysaccharide

A transwell coculture system as we previously described was used to study the effects of MSC MV on protein permeability across HLMVEC monolayer injured by LPS at a concentration of 500 ng/ml (Fig. 2A). FITC-dextran is used as a substitute for albumin to measure the protein permeability of HLMVEC from the upper compartment to the lower compartment and is applied to the upper compartment. As Fig. 2B shows, HLMVEC monolayer damaged by LPS significantly increases the permeability of FITC-dextran through the cell monolayer. The permeability in the LPS group was almost three times higher than in the control group.

### MSC MVs restored protein permeability across HLMVEC injured by LPS independent of S1P receptor-1

Early studies have shown that MSC MV could restore the HLMVEC monolayer permeability insulted by cytomix or LPS [22–24]. In addition, we found that MSC MV significantly increased the expression level of sphingosine-1-phosphokinase 1 (SPHK1) mRNA, indicating that S1P signaling may play a role in the restoration of endothelial permeability by MSC MV. However, the underlining mechanism remains unclear. To explore its role in MSC MV treatment for protein permeability across HLMVEC monolayer injured by LPS, we first blocked the S1P receptor-1 of which S1P activation can enhance barrier integrity [25]

**Fig. 3:**
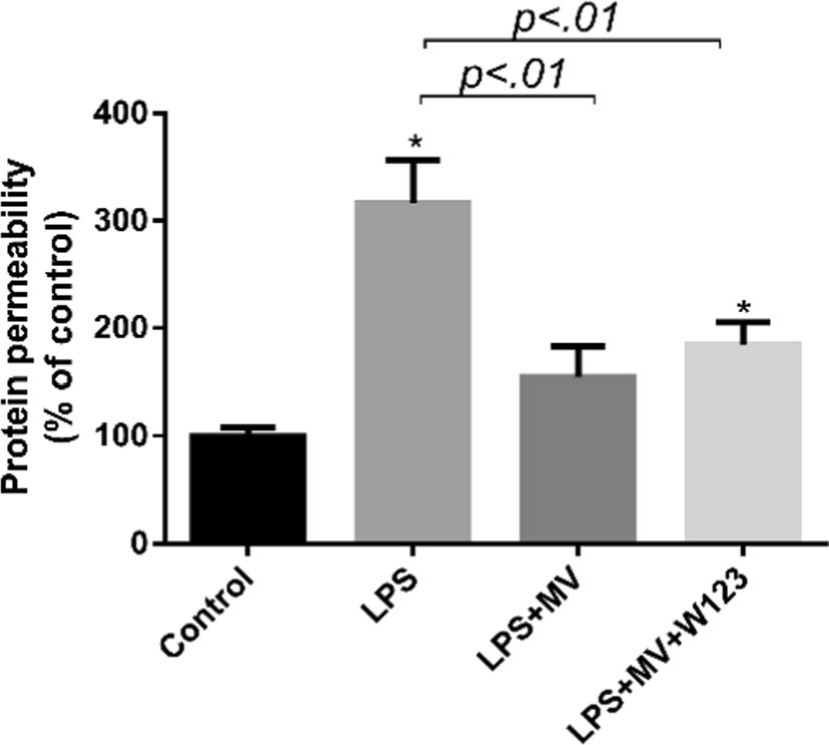
MSC MV restored protein permeability across LPS injured HLMVEC independent of S1P receptor-1. Administration of MSC MV (60 µL) restored protein permeability across LPS (500 ng/ml) injured HLMVEC monolayer over 24 h. Blocking of S1P receptor-1 on HLMVEC by the S1P receptor-1 antagonist (W123, 5 umol/l) showed no impact on the restoration effect of MSC MV on the protein permeability across HLMVEC injured by LPS. Data are presented as mean ± SD, *N* = 4; *p < 0.05 versus control using ANOVA with post hoc Tukey HSD test. MSC, mesenchymal stem cell; MV, microvesicles; LPS, lipopolysaccharide; HLMVEC, human lung microvascular endothelial cells; S1P, sphingosine-1-phosphate; and ANOVA, analysis of variance

HLMVEC monolayer was exposed to 500 ng/ml LPS with or without MSC MV. We used W123 to block the S1P receptor-1 on HLMVEC. After 24 h, the fluorescence intensity of FITC-dextran 70 kDa in the upper chamber and lower chamber was detected by the plate reader. As Fig. 3 shows, exposure to LPS 500 ng/ml further increased protein permeability across HLMVEC to 317% of control. However, when MSC MV (60ul) was added to the top chamber of the transwell coculture system, the protein permeability was significantly decreased to 154% of control. The same treatment effect of MSC MV was observed when adding W123 (185% of control). No significant difference in protein permeability was shown between the LPS + MV and LPS + MV + W123 groups, indicating that the treatment effect of MSC MV on protein permeability across HLMVEC injured by LPS was independent of S1P receptors on HLMVEC.

### MSC MV restored cytoskeleton protein F-action, tight junction protein ZO-1 and adherens junction protein VE-cadherin and β-catenin of LPS injured HLMVEC via independent of S1P receptor-1

**Fig. 4:**
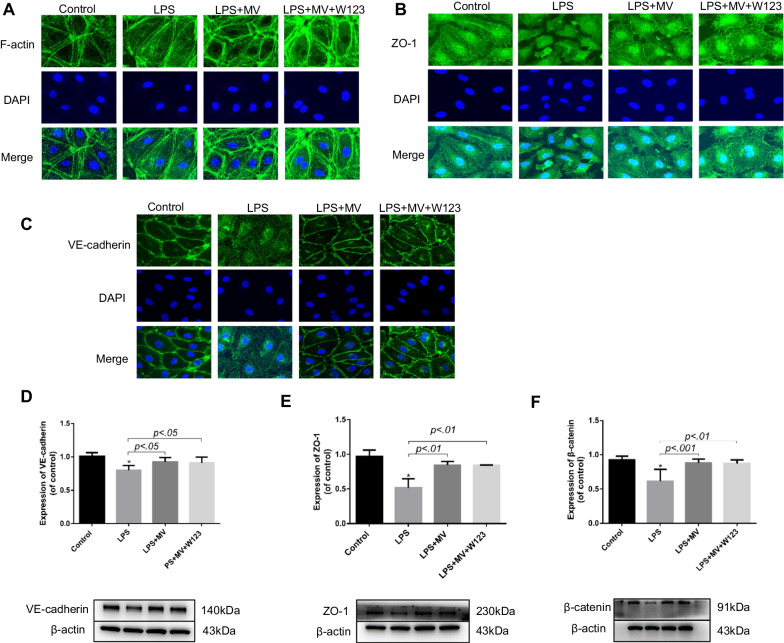
MSC MV restored the rearrangement of the cytoskeleton protein F-action, the loss of tight junction protein ZO-1, and adherens junction protein VE-cadherin and β-catenin of LPS injured HLMVEC independent of S1P receptor-1. HLMVEC were seeded on cell slides and stained with **A** phalloidin (green) for F-actin**, B** Goat anti-rabbit IgG H&L (Alexa flour®488) (green) for ZO-1, and **C** Goat anti-mouse IgG H&L (Alexa flour®488) (green) for VE-cadherin. F-actin staining in HLMVEC of the control group showed a typical peripheral distribution, and VE-cadherin and ZO-1 staining were strong at the junctions between cells. After being exposed to LPS for 24 h, F-actin of cells reorganized into the center of the cell to form “actin stress fibers,” and the staining of cell adhesion junction protein VE-cadherin and cell tight junction protein ZO-1 was lost, resulting in an increase in cell pore size, which may be the reason for the increased protein permeability. The administration of MSC MV largely prevented the reorganization of cytoskeleton protein F-actin into “actin stress fibers” and restored the staining of VE-cadherin and ZO-1 between HLMVEC cells damaged by LPS. Images are representative for each condition run in triplicates. DAPI (blue) was used to stain the cell nuclei. **D** Western blot analyses showed that the loss in VE-cadherin total protein levels with LPS injury was partially restored by MSC MV treatment. **E** Western blot analyses showed that the loss in ZO-1 total protein levels with LPS injury was partially restored by MSC MV treatment. **F** Western blot analyses showed that the loss in β-catenin total protein levels with LPS injury was also partially restored by MSC MV treatment. The therapeutic effect of MSC MVs on the cytoskeleton structure and cell junction proteins was not abolished by adding S1P receptor-1 antagonist W123. Data are presented as mean ± SD, *N* = 3, *p < 0.05 versus control using ANOVA with post hoc Tukey HSD test. Abbreviations: MSC, mesenchymal stem cell; MV, microvesicles; LPS, lipopolysaccharide; HLMVEC, human lung microvascular endothelial cells; S1P, sphingosine-1-phosphate; and ANOVA, analysis of variance

To better understand the role of S1P in MSC MV treatment for endothelial permeability, we blocked the S1P receptor on endothelial cells and used immunofluorescence and western blotting to study the distribution of the cytoskeletal protein F-actin, the adhesion junction protein VE-cadherin and β-catenin, and the tight junction protein zonula occludens-1 (ZO-1). The results showed that MSC MV significantly improved the rearrangement of cytoskeletal protein F-actin into “actin stress fibers” in HLMVEC damaged by LPS (Fig. 4A). Moreover, MSC MV treatment significantly prevented the loss of the adhesion-associated protein VE-cadherin and β-catenin (Fig. 4C, D, F), as well as tight junction protein ZO-1 (Fig. 4B, E) in LPS injured HMVEC. The blockage of S1P receptor on HLMVEC had no impact on the effect of MSC MV in F-action, ZO-1 and adherens junction protein VE-cadherin and β-catenin and further demonstrated that the therapeutic effect of MSC MV on protein permeability was not dependent on S1P receptor.

### MSC MV increased SPHK1 and intracellular S1P levels in LPS injured HLMVEC

**Fig. 5:**
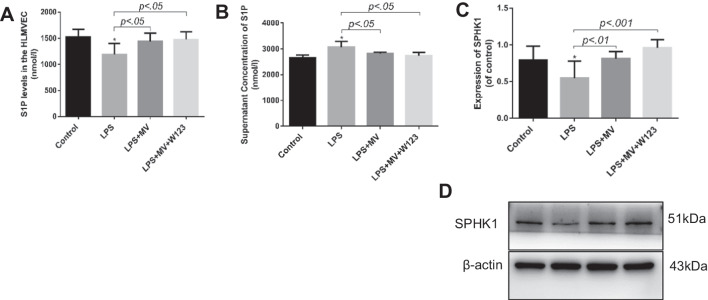
MSC MV increased SPHK1 and intracellular S1P levels in LPS injured HLMVEC. S1P levels in HLMVEC and in the cell supernatant at were detected by ELISA. **A** MSC MV significantly increased S1P secretion in HLMVEC at 24 h. **B** However, MSC MV significantly decreased the S1P expression in the supernatant of HLMVEC at 24 h. The effect of MSC MV on S1P levels in HLMVEC and in the cell supernatant was not abolished by the addition of W123. **C, D **By western blotting analysis, MSC MV treatment increased SPHK1 expression in LPS injured HLMVEC, and the increase in SPHK1 was not affected by the addition of W123. Data are presented as mean ± SD, *N* = 4–9. *p < 0.05 versus control using ANOVA with post hoc Tukey HSD test. MSC, mesenchymal stem cell; MV, microvesicles; HLMVEC, human lung microvascular endothelial cells; SPHK1, sphingosine kinases 1; S1P, sphingosine-1-phosphate; and ANOVA, analysis of variance

Due to the presence of mRNA, microRNA proteins/peptides, lipids and organelles, MSC MV has been shown to be biologically active. In our previous study, we also found that the expression level of sphingosine-1-phosphokinase 1 (SPHK1) mRNA in damaged HLMVEC increased significantly after MSC MV treatment [22]. To further understand the role of S1P in the therapeutic effects of MSC MVs, we measured SPHK1 and S1P in HLMVEC as well as S1P in HLMVEC supernatant. We found that as compared with LPS group, the expression of SPHK1 protein in HLMVEC in both the LPS + MV group and LPS + MV + W123 groups significantly increased. However, there was no significant difference between the LPS + MV group and LPS + MV + W123 groups (Fig. 5C, D). Moreover, ELISA results showed that as compared with the LPS group, the level of S1P in HLMVEC in both the LPS + MV and LPS + MV + W123 groups increased and the levels of S1P in HLMVEC supernatant decreased statistically. But no significant difference was found in the S1P levels in HLMVEC and in HLMVEC supernatant between the LPS + MV and LPS + MV + W123 groups (Fig. 5A, B). Given that the therapeutic effect of MSC MV on HLMVEC monolayer permeability was not abolished by the blockage of S1P receptor-1 and the generation of S1P in HLMVEC was increased, we could concluded that MSC MV improved protein permeability across LPS injured HLMVEC partly by increasing S1P level in HLMVEC.

### MSC MV promoted Ca^2+^ mobilization and activated Rac1 pathway in LPS injured HLMVEC by increasing its intracellular S1P level

Rac activity is required for S1P-induced adherens junction assembly and cytoskeleton rearrangement [26]. A previous study found that increasing the level of S1P in the cytoplasm of endothelial cells through photolysis could enhance intracellular Ca^2+^ mobilization, thereby inducing the activation of Rac1/IQGAP1 pathway, causing the rearrangement of cytoskeletal protein, the adherens junction protein-VE-cadherin, β-catenin, thereby reducing vascular endothelial permeability. The administration of S1P receptor-1 antagonist can block the improvement of vascular permeability caused by exogenous S1P, but has no effect on the improvement of vascular permeability caused by the increase in intracellular S1P level caused by photolysis [27]. Therefore, the accumulation of S1P in cells can increase the intracellular calcium ion mobilization, thereby directly activating the Rac1 pathway and enhancing the endothelial barrier function.

**Fig. 6:**
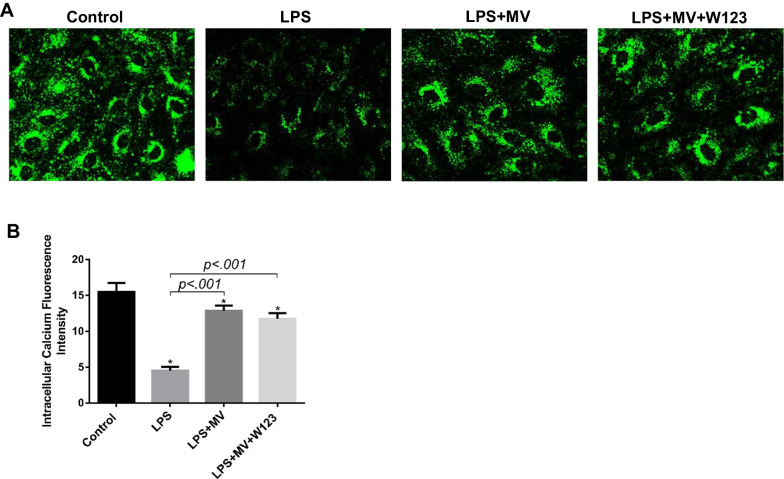
MSC MV promoted Ca^2+^ mobilization in injured HLMVEC by increasing its intracellular S1P. **A** Fluorescence was observed after HLMVEC treated with Ca^2+^ probe Flu-3AM by laser confocal. **B** Intracellular calcium fluorescence intensity in different groups was analyzed by ImageJ. Data are presented as mean ± SD, *N* = 3. *p < 0.05 versus control using ANOVA with post hoc Tukey HSD test. MSC, mesenchymal stem cell; MV, microvesicles; HLMVEC, human lung microvascular endothelial cells; S1P, sphingosine-1-phosphate; and ANOVA, analysis of variance

**Fig. 7:**
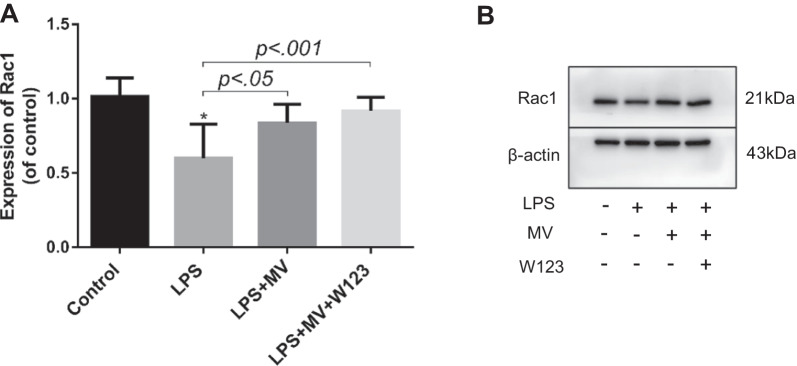
MSC MV activated Rac1 pathway in injured HLMVEC by increasing its intracellular S1P. Rac1 is a downstream molecule of the intracellular Ca^2+^ signaling pathway. The increase in intracellular S1P levels could activate downstream Rac1 through the mobilization of intracellular Ca^2+^. **A** Western blotting analysis showed that administration of MSC MV increased the activation of Rac1 pathway in HLMVEC injured by LPS. The addition of W123 had not abolished the effect of MSC MV on Rac1 activation. **B** The representative image of Rac1. Data are presented as mean ± SD, N = 7. *p < .0.05 versus control using ANOVA with post hoc Tukey HSD test. MSC, mesenchymal stem cell; MV, microvesicles; HLMVEC, human lung microvascular endothelial cells; S1P, sphingosine-1-phosphate; and ANOVA, analysis of variance

To explore the downstream signaling molecular of S1P, we used laser confocal to measure the Ca^2+^ mobilization and Rac activation in HLMVEC. Laser confocal results (Fig. 6A, B) showed that LPS impaired the Ca^2+^ mobilization in HLMVEC dramatically. MSC MV treatment significantly improved the mobilization of Ca^2+^ in LPS injured HLMVEC. And this therapeutic effect of MSC MV was not affected by adding S1P receptor-1 antagonist W123. Meanwhile, as compared with LPS group, Rac1 activation in HLMVEC in both the LPS + MV and LPS + MV + W123 groups significantly increased, but no difference was found in the activation of Rac1 in HLMVEC between the LPS + MV and LPS + MV + W123 groups (Fig. 7A, B). The results implied that MSC MV promoted Ca^2+^ mobilization and activated Rac1 pathway in LPS injured HLMVEC by increasing intracellular S1P independent of S1P receptor-1.

## Discussion

ARDS is a diffuse injury of pulmonary capillary endothelium and alveolar epithelium which result in alveolar-capillary membrane disruption [1, 2]. Previous study has shown that MSC MV could restore endothelial function [28]. However, the mechanism of MSC MV stabilizing endothelial permeability is not yet detailed [29]. In the present study, what are mainly found is summarized as follows: (a) MSC MV restored protein permeability across HLMVECs monolayer injured by LPS; (b) MSC MV prevented the rearrangement of the cytoskeleton protein F-actin and the loss of tight junction proteins and adhesion junction proteins in HLMVEC damaged by LPS; (c) MSC MV increased the expression level of SPHK1 in LPS injured HLMVEC; (d) MSC MV increased S1P concentration in HLMVEC and decreased in its supernatant; (e) Ca^2+^ release was increased after MSC MV treatment; (f) MV also increased the Rac1 activation in LPS injured HLMVEC; and (g) those effects of MSC MV were independent of S1P receptor-1 on HLMVEC.

Mesenchymal stem cells (MSCs) have been found to release microvesicles which have shown biological function which is similar to its parent stem cells [17, 31, 32]. MSC MV has been reported to exert therapeutic effects in several preclinical models [21, 30, 32–35]. Recently, increasing evidence suggests that MSC MV also could stabilize the endothelial barrier. Our previous study has shown that MSC MV increased Ang1 secretion in injured HLMVEC to restore protein permeability across primary cultures of injured HLMVEC monolayer [22]. In this present study, we demonstrated again that MSC-derived microvesicles could restore lung permeability by increasing the expression of intercellular junction protein and preventing “actin stress fiber” formation.

More importantly, our data suggest that S1P signaling played an important role in MSC MV to restore lung permeability. Sphingosine-1-phosphate (S1P) is a sphingolipid produced by the phosphorylation of sphingosine, which is a sphingomyelin catabolism catabolized by sphingosine kinase (SPHK). S1P can be used as an extracellular ligand to bind to the S1P receptor on the cell membrane, or as a “second messenger” in the cell, thereby exerting its multiple biological effects in regulating vascular permeability, promoting cell proliferation, inhibiting apoptosis, mediating cell rolling, and regulating inflammation [25]. In this present study, we observed that the therapeutic effect of MSC MV was associated with increase in intracellular S1P and it was not abolished when adding S1PR1 antagonist. The finding implies that S1P may function intracellularly as a second messenger rather than activate and signal through S1P receptor-1 present on the surface of the endothelium.

This is consistent with the findings from previous studies [27]. Usatyuk et al. [27] reported that intercellular S1P could exert therapeutic effects as a second messenger which was independent of S1PR1. S1P released intracellularly directly induced calcium (Ca^2+^) release from the endoplasmic reticulum, activated MAPKs and Rac1/IQGAP1, further caused the redistribution of cytoskeletal, focal adhesion and tight junction proteins and thereby improved endothelial barrier function. To further characterize the downstream molecular of S1P signaling in the therapeutic effect of MSC MV, we next explored the Ca^2+^ release in this present study. We found that MSC MV increased the intracellular Ca^2+^ level in injured HLMVEC. Moreover, the activation of Rac1 elevated significantly in injured HLMVEC when treated with MSC MV. Interestingly, Ca^2+^ release and Rac1 activation were not significantly impacted when adding S1P receptor-1 antagonist, further supporting the role for intracellular S1P in modulating signaling pathways independent of S1P receptors in the injured endothelium.

The mechanism for MSC MV to increase the intracellular S1P in injured HLMVEC is not fully understood. In a recent study, Chuan Xiang et al. [36] found that MSC MVs enriched high level S1P compared with MSC, which is consistent with previous results that MV is 2–3 times more potent in cholesterol, phosphatidylserine and glycosphingolipid than cells [37]. It has been proved that MSC MV could stabilize endothelial permeability by the transfer of its content to the injured endothelial [38]. Therefore, the therapeutic effect of MSC MV may be related to the transfer of S1P from MV to the injured endothelial. Moreover, SPHK1 may be responsible for the S1P enrichment in the injured HLMVEC as well. S1P is generated intracellularly from the phosphorylation of sphingosine mainly by SPHK1. There is accumulating evidence that activation of sphingosine kinase 1 (SPHK1) is an important element in intracellular S1P signaling cascades in endothelium [39]. In our previous study, we found that MSC MVs increased the expression of SPHK1 in injured HLMVEC after treated with MSC MV [22]. This was confirmed again in this present study. The increase in SPHK1 expression in HLMVEC could be one of the contributors to the increase in the S1P level in injured HLMVEC which further enhanced the endothelial barrier permeability. Besides, this study found that the S1P level in HLMVEC culture medium increased significantly by MSC MV treatment. Exogenous S1P can be converted to intracellular S1P in endothelium [40]. This may be one of the reasons for the increase in intracellular S1P level in the injured HMLVEC.

The limitations of our experimental design still need further study: ① We used LPS to establish an endothelial cell permeability model, which cannot fully simulate the complex microenvironment of ARDS endothelial permeability. ② The mechanism for MSC MV to increase the intracellular S1P in injured HLMVEC remains unclear. MSC MV itself is rich in S1P, SPHK1 mRNA, and other mRNA, microRNA and organelles which could cause the increase in intracellular S1P level. Moreover, endothelium can convert extracellular S1P to intracellular S1P which could also be one reason for the increase in intracellular S1P.

## Conclusions

Our results presented here demonstrate that MSC MV restored protein permeability across HLMVEC in part by maintaining intercellular junctions and preventing “actin stress fiber” formation, partly through the intracellular S1P signaling pathway independent of S1P receptor-1.

## Data Availability

All data generated or analyzed during this study are included in this published article.

## Abbreviations

MSC: Mesenchymal stem cell
MV: Microvesicles
HLMVEC: Human lung microvascular endothelial cells
S1P: Sphingosine-1-phosphate
NTA: Nanoparticles tracking technology
LPS: Lipopolysaccharide
ELISA: Enzyme-linked immunosorbent assay
FBS: Fetal bovine serum
PBS: Phosphate-buffered saline
